# A Pan-Cancer Analysis of microRNA Tissue Specificity and Its Association with Dysregulation

**DOI:** 10.3390/ijms27188153

**Published:** 2026-09-13

**Authors:** Aly Ismailov, Alexey Belogurov, Alena Evpak, Maria Poptsova

**Affiliations:** 1 Centre for Biomedical Research and Technology, HSE University, 109028 Moscow, Russia; ismailov.aly@gmail.com; 2 Laboratory of Hormonal Regulation Proteins, Shemyakin and Ovchinnikov Institute of Bioorganic Chemistry Russian Academy of Sciences (RAS), 117997 Moscow, Russiaalena.st97@gmail.com (A.E.); 3 Department of Biological Chemistry, Russian University of Medicine, Ministry of Health of Russian Federation, 127473 Moscow, Russia

**Keywords:** miRNA, pan-cancer, cancer, tissue-specificity, differential expression, dysregulation, oncomiRs

## Abstract

MicroRNAs are frequently dysregulated in cancer, yet how their tissue specificity is remodeled during malignant transformation remains poorly characterized. Here, we systematically quantified the tissue specificity of miRNAs across normal (GTEx) and cancer (TCGA) tissues using the Tau index as the primary metric and the Gini index for independent validation. Our analysis revealed a negative association between tissue specificity in healthy tissues and changes in specificity during malignant transformation, indicating that miRNAs with higher tissue specificity in normal tissues tend to undergo greater loss of specificity in cancer. To robustly define dysregulation, we combined two independent analyses: a binomial test over per-project differential expression across 17 matched normal tissues within the TCGA cohort, and a TCGA–GTEx pan-tissue comparison of mean expression. The change in specificity (ΔTau) separated up- from downregulated miRNAs, showing moderate agreement with the binomial signal and a strong correlation with the expression-based contrast. Finally, we identified six miRNAs that lose tissue specificity upon transformation while remaining consistently upregulated (miR-519a-5p, miR-512-3p, miR-522-3p, miR-105-5p, miR-935, miR-1269a). Functional analysis of their experimentally validated and predicted targets showed significant enrichment for converging on core oncogenic programs. Collectively, integrating specificity dynamics with dysregulation evidence pinpoints candidate miRNAs with coordinated, cancer-relevant regulatory roles and highlights those with favorable tissue-specificity profiles that may warrant further investigation as potential therapeutic candidates.

## 1. Introduction

MicroRNAs (miRNAs) are small non-coding RNAs that act as key post-transcriptional regulators of gene expression and play essential roles in the maintenance of cellular identity, differentiation, and tissue homeostasis. By fine-tuning the expression of multiple target genes simultaneously, miRNAs coordinate diverse biological processes, including development, proliferation, apoptosis, and immune responses. Dysregulation of miRNA expression is a well-established hallmark of cancer and contributes to numerous oncogenic processes, including uncontrolled proliferation, invasion, metastasis, angiogenesis, immune evasion, and cellular plasticity [1].

Over the past two decades, extensive studies have identified tumor-associated miRNAs and established their potential utility as diagnostic biomarkers, prognostic indicators, and therapeutic targets [1]. However, most pan-cancer investigations have focused primarily on differential expression analysis, emphasizing whether specific miRNAs are upregulated or downregulated in tumors relative to normal tissues [2,3,4]. While this approach has yielded important insights, it does not capture an important aspect of miRNA biology, that is tissue specificity. In normal tissues, tissue-specific miRNAs help stabilize lineage-associated transcriptional states and suppress alternative differentiation programs, thereby maintaining cellular phenotype and functional specialization [5,6,7]. Disruption of these regulatory constraints may represent a critical feature of malignant transformation, enable dedifferentiation and increase cellular plasticity. Nevertheless, the extent to which loss of tissue specificity contributes to recurrent pan-cancer miRNA deregulation remains poorly understood.

In this study, we performed an integrative analysis of two major cohorts: healthy tissues from the Genotype-Tissue Expression (GTEx) project and tumor samples from TCGA, TARGET, CGCI, and CPTAC, hereafter collectively referred to as the TCGA cohort for simplicity. Tissue specificity was quantified using the Tau index across tissues in both cohorts, with the Gini index used as an independent validation metric. To identify miRNAs associated with malignant transformation, we applied two complementary analytical approaches. First, differential expression analysis was performed within individual cancer projects by comparing tumor samples with matched normal tissues where available. Second, miRNA expression patterns and tissue specificity profiles were compared between the GTEx and TCGA cohorts. The results obtained from both approaches were integrated to identify robust miRNA signatures consistently associated with malignant transformation. This analysis revealed six miRNAs that lost their tissue specificity upon malignant transformation, shifting from tissue-restricted expression patterns in healthy tissues to widespread activation across tissues in cancer samples. Enrichment analysis of experimentally validated and predicted targets of these miRNAs supported their involvement in cancer-related biological processes, highlighting their potential as candidates for antagomir-based therapeutic strategies due to their restricted expression in normal tissues and broad activation across tumor types.

## 2. Results

### 2.1. Tissue Specificity of miRNAs in Cancer and Healthy Cohorts

**Figure 1:**
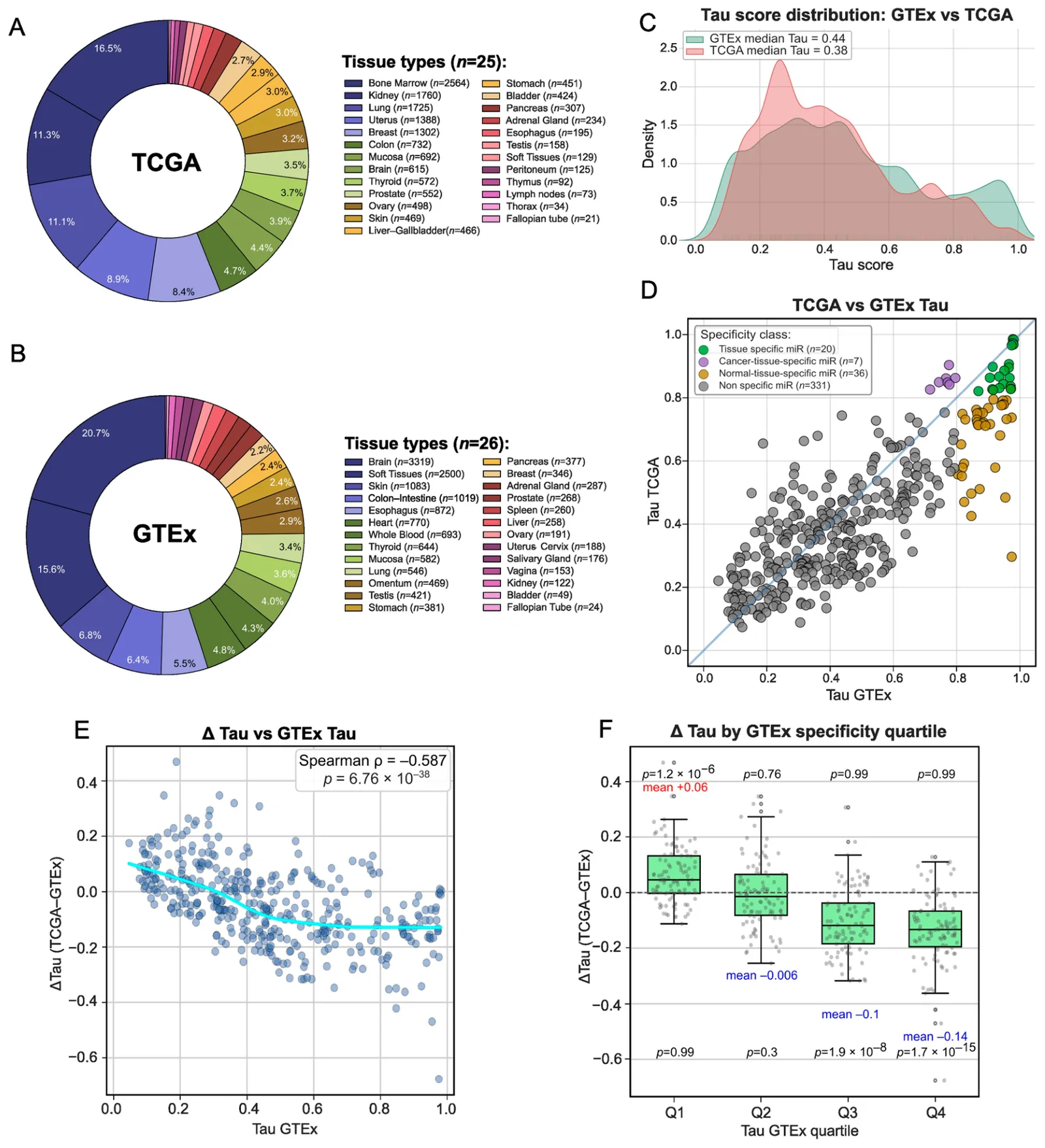
Overview of the datasets and tissue specificity analysis. (**A**) Distribution of healthy tissue types in the GTEx cohort. (**B**) Distribution of cancer tissue types in the TCGA cohort. (**C**) Distribution of tissue specificity (Tau) scores across tissues in the GTEx and TCGA cohorts. (**D**) Scatter plot of tissue specificity (Tau) scores for individual miRNAs in the GTEx (*x*-axis) and TCGA (*y*-axis) cohorts. Using the commonly applied Tau threshold of 0.8, miRNAs were classified into four categories: tissue-specific (green), cancer-tissue-specific (purple), normal-tissue-specific (yellow), and non-specific (gray). Points above the diagonal solid line indicate a positive shift in tissue specificity (positive ΔTau), whereas points below the line indicate a negative shift (negative ΔTau). (**E**) Scatter plot of ΔTau (TCGA − GTEx) against GTEx Tau. An inverse correlation was observed between tissue specificity in normal tissues and the change in tissue specificity during malignant transformation (Spearman’s ρ = −0.59, *p* = 6.76 × 10^−38^), with miRNAs exhibiting higher tissue specificity in GTEx tending to show a greater loss of specificity in TCGA. The solid curve represents the LOWESS-smoothed trend. (**F**) Boxplots showing the distribution of ΔTau values across GTEx Tau quartiles. Binomial tests were performed under the null hypothesis of a random distribution of miRNAs above and below ΔTau = 0 (*p* = 0.5). miRNAs in Q1 showed a significant tendency toward positive ΔTau values (*p* = 1.2 × 10^−6^), whereas Q2 showed no significant deviation in either direction. miRNAs in Q3 and Q4 showed significant shifts toward negative ΔTau values (*p* = 1.9 × 10^−8^ and 1.7 × 10^−15^, respectively).

To enable a comparable analysis between the GTEx and cancer cohorts, miRNA expression profiles were stratified according to tissue of origin, resulting in 25 tissue groups in the cancer cohort and 26 corresponding tissue groups in the GTEx dataset (Figure 1A,B). For the analysis of tissue specificity, we used two well-established specificity indices, Tau [8] and Gini. Comparative analysis demonstrated a high concordance between the two indices, both across TCGA tissues (Spearman’s ρ = 0.96) and across GTEx tissues (Spearman’s ρ = 0.94). The Tau index showed a more uniform distribution of values, whereas the Gini index exhibited a pronounced right-skew (Supplementary Figure S1A). Therefore, the Tau index was used for subsequent analyses. We applied the specificity threshold of Tau = 0.8, as suggested in the literature [9]. This threshold approximately corresponds to more than 90% of total CPM expression being concentrated in four or fewer tissues, which we considered a sufficiently stringent criterion for tissue specificity. Notably, an analogous criterion for the Gini index yielded a threshold of 0.5 (Supplementary Figure S1B), which is also consistent with previously published values [10]. The overall distribution of tissue specificity scores (Tau) demonstrated comparable patterns between normal and tumor tissues, with median Tau values of 0.44 and 0.38 in GTEx and TCGA cohorts, respectively (Figure 1C). Using a Tau threshold of 0.8, miRNAs were classified into four categories according to their tissue specificity patterns in normal and cancer tissues. Tissue-specific miRNAs were defined as miRNAs with Tau values above the threshold in both normal and cancer tissues, indicating that they retain tissue-specific expression despite malignant transformation. Cancer-tissue-specific miRNAs were characterized by Tau values below the threshold in normal tissues but above the threshold in cancer tissues, indicating that they acquire tissue-specific expression following malignant transformation. Conversely, normal-tissue-specific miRNAs showed tissue-specific expression in normal tissues (Tau > 0.8) but lost this specificity in cancer tissues (Tau ≤ 0.8). Finally, non-specific miRNAs had Tau values below the threshold in both normal and cancer tissues, indicating a lack of pronounced tissue-specific expression in either context (Figure 1D). To validate the consistency of the tissue-specificity analysis, we compared the highly tissue-specific miRNAs identified in our analysis with data from miRNATissueAtlas 2025 [11]. This comparison demonstrated a high degree of concordance. Specifically, the highly tissue-specific miRNAs miR-302a-5p, miR-302a-3p, miR-302d-3p, miR-302b-3p, miR-215-3p, miR-1298-3p, and miR-211-5p (Supplementary Figure S2A–G), which had Tau index values > 0.9 in our analysis, also showed high tissue specificity index scores (TSI > 0.9), with expression predominantly detected in similar tissues. The overall correlation between GTEx Tau and miRNATissueAtlas TSI was strong (Pearson’s r = 0.87, Spearman’s ρ = 0.84; Supplementary Figure S2H).

Although the Tau distributions in GTEx and TCGA appeared broadly similar at first glance, further analysis revealed a clear pattern of attenuation of tissue specificity. Specifically, miRNAs with higher GTEx specificity preferentially exhibited negative ΔTau values, indicating a loss of tissue specificity (Figure 1E,F). This trend was also reflected in both the number and direction of tissue-specific miRNA calls in the cancer-tissue-specific and normal-tissue-specific groups. Normal-tissue-specific calls significantly outnumbered cancer-tissue-specific calls (36 versus 7; binomial *p* = 4.5 × 10^−6^; Supplementary Table S1). Moreover, within the normal-tissue-specific group, miRNAs with negative ΔTau values overwhelmingly predominated (54 versus 2; binomial *p* = 2.2 × 10^−14^; Supplementary Table S1). A similar, although less pronounced, pattern was observed in the cancer-tissue-specific group, where most miRNAs also exhibited negative ΔTau values (18 versus 9; binomial *p* = 0.06; Supplementary Table S1).

In contrast, miRNAs with low tissue specificity in normal tissues (i.e., those in the lowest Tau quartile, Q1) tended to exhibit positive ΔTau values, although the magnitude of this shift was modest (mean ΔTau +0.05; binomial *p* = 1.2 × 10^−6^; Figure 1F), indicating a modest gain in specificity among broadly expressed normal-tissue miRNAs. miRNAs in the second quartile (Q2) showed no pronounced change in tissue specificity, whereas those in Q3, and particularly Q4, demonstrated a significant loss of specificity (Figure 1F). Comparable results were obtained using the Gini metric (Supplementary Table S1). Altogether, these results support a tendency toward loss of tissue specificity during malignant transformation among miRNAs that are highly tissue-specific in healthy tissues.

### 2.2. Dysregulated miRNAs in Cancer

**Figure 2:**
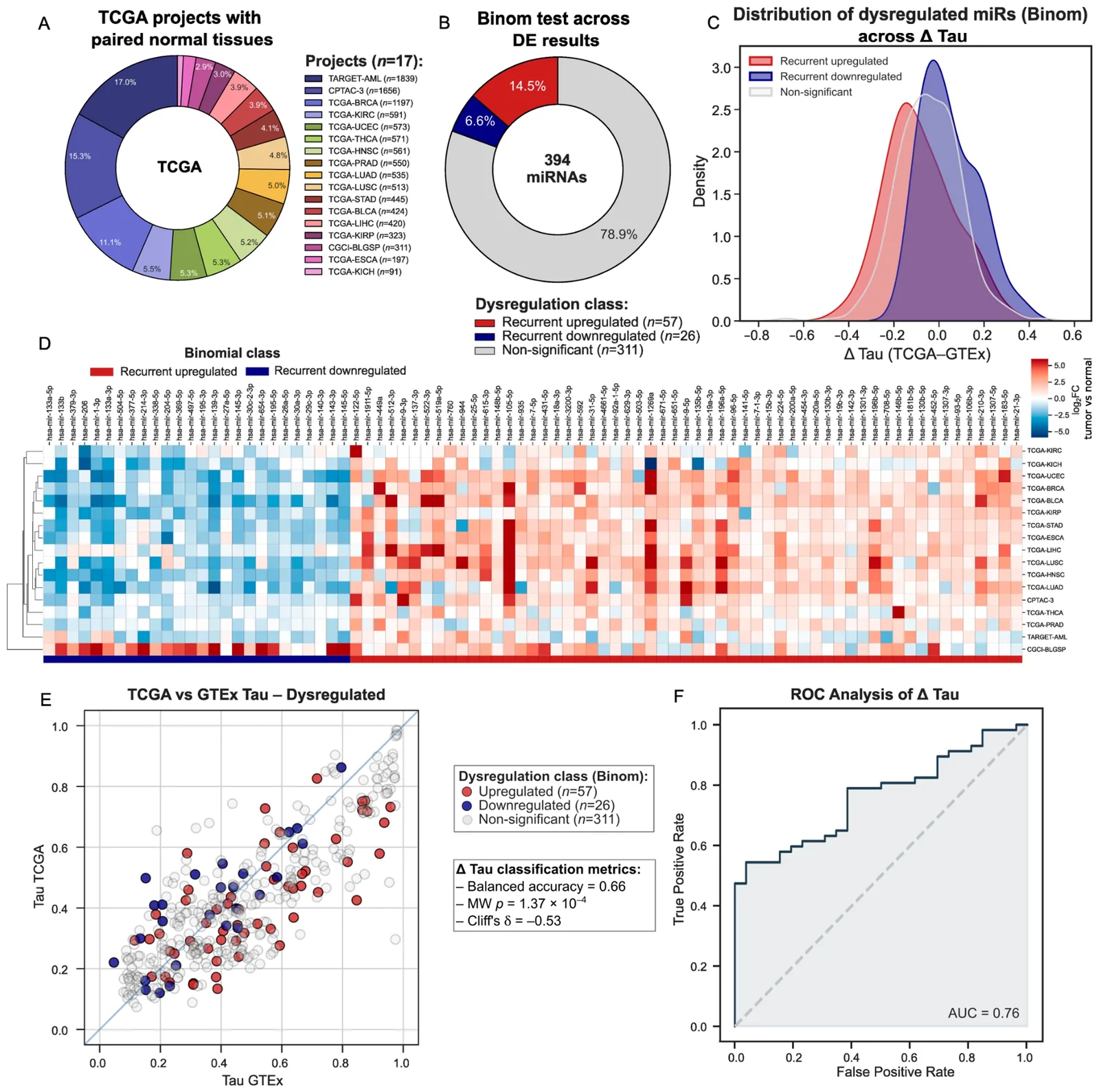
Identification of recurrently dysregulated miRNAs and their association with changes in tissue specificity. (**A**) Distribution of cancer projects within the TCGA cohort with available matched normal tissues. (**B**) Results of the directed binomial test following differential expression (DE) analysis across 17 cancer cohorts. Among 394 analyzed miRNAs, 311 (78.9%) did not demonstrate consistent dysregulation across cancer cohorts, whereas 57 miRNAs (14.5%) were recurrently upregulated and 26 miRNAs (6.6%) were recurrently downregulated. (**C**) Distribution of ΔTau values for dysregulated miRNAs. Recurrently upregulated miRNAs showed a shift toward negative ΔTau values, indicating reduced tissue specificity in cancer, whereas recurrently downregulated miRNAs tended toward positive ΔTau values. (**D**) Heatmap showing log_2_ fold-change values for 83 significantly dysregulated miRNAs across 17 cancer cohorts included in the differential expression analysis. (**E**) Scatter plot comparing tissue specificity scores (Tau) between GTEx and cancer cohorts. Each point represents an individual miRNA and is colored according to its recurrent differential expression status (upregulated, downregulated, or non-significant). The blue diagonal represents equal tissue specificity between normal and cancer cohorts, miRNAs below the diagonal exhibit reduced tissue specificity in cancer (negative ΔTau), whereas miRNAs above the diagonal exhibit increased tissue specificity (positive ΔTau). ΔTau showed moderate discrimination between recurrently upregulated and downregulated miRNAs (balanced accuracy = 0.66, MW U test *p* = 1.37 × 10^−4^; Cliff’s δ = −0.53). (**F**) Receiver operating characteristic (ROC) curve evaluating the ability of ΔTau to discriminate between recurrently upregulated and recurrently downregulated miRNAs. ΔTau demonstrated moderate discriminatory performance between the two classes (ROC-AUC = 0.76). Dashed diagonal line demonstrates performance of random classifier.

Differential expression analysis was performed independently across 17 cancer projects with available matched tumor and normal samples (Figure 2A). Complete differential expression results are available in the Supplementary Table S2. According to the binomial test (see Methods), a large fraction of miRNAs (*n* = 311, 78.9%) did not show a significant enrichment toward either up- or downregulation among significant differential expression events. Among all analyzed miRNAs, 14.6% (*n* = 57) were classified as recurrently upregulated, while 6.6% (*n* = 26) were classified as recurrently downregulated across cancer types (Figure 2B).

The distribution of dysregulated miRNAs along the ΔTau axis showed a modest shift in upregulated miRNAs toward negative ΔTau values, indicating a tendency to lose tissue specificity, whereas downregulated miRNAs were shifted toward positive ΔTau values, reflecting increased tissue specificity (Figure 2C). The structure of dysregulation changes across TCGA projects is further illustrated in the heatmap (Figure 2D), which includes only statistically significant events (|log_2_FC| > 1, FDR < 0.01 for differential expression and *p* < 0.05 for the binomial test). Notably, the Burkitt Lymphoma Genome Sequencing Project (CGCI-BLGSP) shows a distinct inverted expression pattern. In this cohort, most miRNAs that are typically downregulated across other cancer types show significant upregulation (Figure 2D). ΔTau values differed significantly between recurrently upregulated and downregulated miRNAs (Mann–Whitney U test, *p* = 1.37 × 10^−4^), showing a moderate effect size (Cliff’s δ = −0.53). ΔTau demonstrated meaningful discriminatory ability between these two classes (ROC-AUC = 0.76). Using ΔTau = 0 as a threshold, the classification achieved a balanced accuracy of 0.66 (Figure 2C). The ΔGini discriminator demonstrated similar classification performance, with a balanced accuracy of 0.66, a Mann–Whitney U test *p*-value of 3.33 × 10^−4^, and a Cliff’s δ of −0.49 (Supplementary Figure S3A–C).

**Figure 3:**
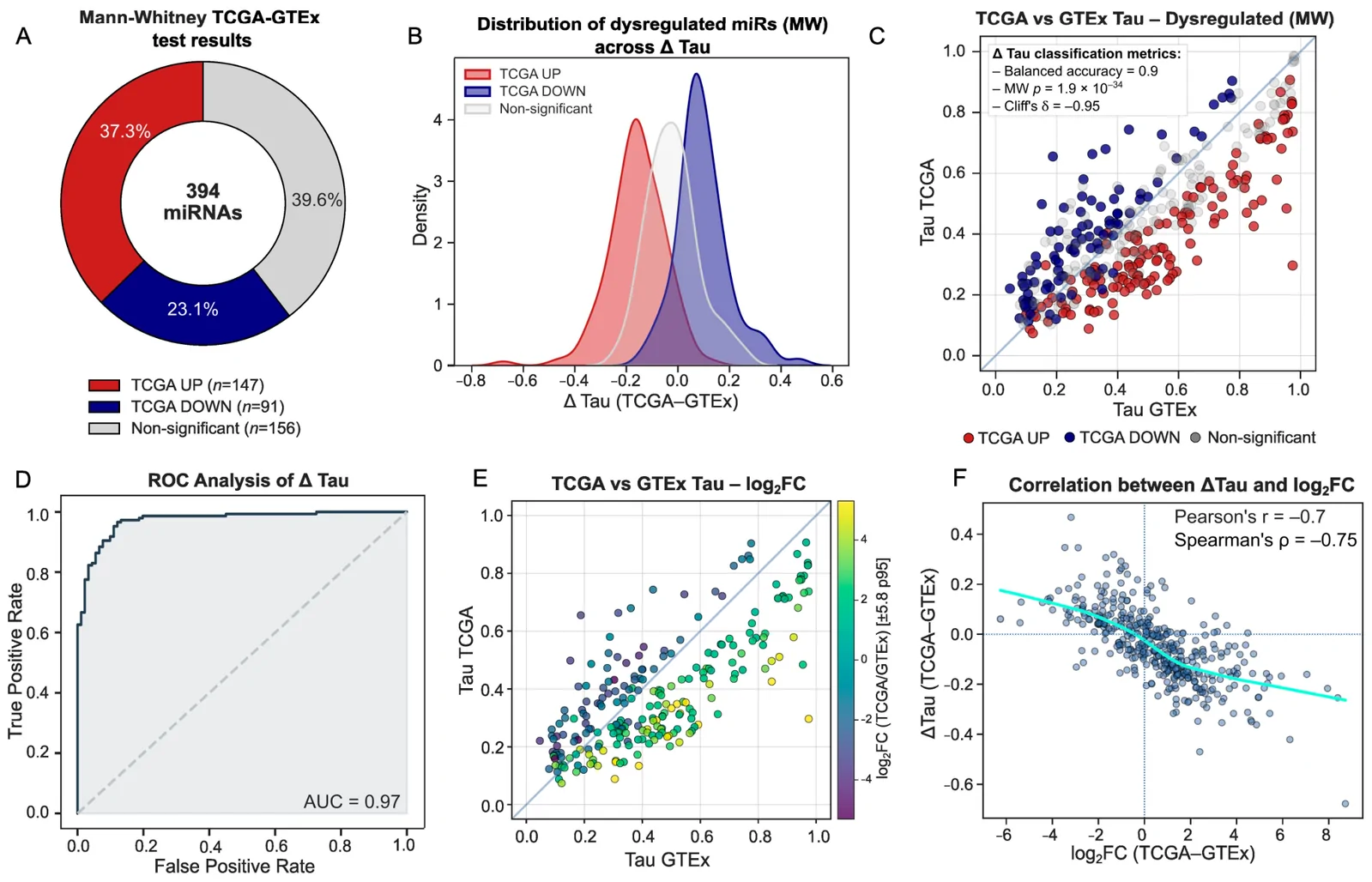
Association between cancer-associated expression shifts and tissue specificity dynamics. (**A**) Distribution of miRNAs based on differential CPM expression between GTEx and TCGA cohorts across tissue types (Mann–Whitney FDR < 0.01; |log_2_FC| > 1). Among 394 analyzed miRNAs, 147 (37.3%) showed increased expression in cancer tissues (TCGA-upregulated), 91 (23.1%) showed increased expression in GTEx tissues (GTEx-upregulated), and 156 (39.6%) did not demonstrate significant expression differences. (**B**) Distribution of ΔTau values among the three miRNA classes (TCGA-upregulated, GTEx-upregulated, and non-significant miRNAs). Compared with recurrent dysregulation analysis, expression-based classification demonstrated a stronger separation of ΔTau distributions, with cancer-upregulated miRNAs preferentially showing reduced tissue specificity. (**C**) Scatter plot comparing tissue specificity scores (Tau) between GTEx and cancer cohorts. Each point represents an individual miRNA and is colored according to its expression-based differential expression status (TCGA-upregulated, GTEx-upregulated, or non-significant). The diagonal represents equal tissue specificity between normal and cancer cohorts; miRNAs below the diagonal exhibit reduced tissue specificity in cancer (negative ΔTau), whereas miRNAs above the diagonal exhibit increased tissue specificity (positive ΔTau). ΔTau showed strong discrimination between TCGA-upregulated and GTEx-upregulated miRNAs (balanced accuracy = 0.9; Mann–Whitney U test, *p* = 1.9 × 10^−34^; Cliff’s δ = −0.95). (**D**) Receiver operating characteristic (ROC) curve evaluating the ability of ΔTau to distinguish between TCGA-upregulated and GTEx-upregulated miRNAs. ΔTau demonstrated strong discriminatory performance between the two classes (ROC-AUC = 0.97). (**E**) Scatter plot comparing Tau scores between GTEx and cancer cohorts, with individual miRNAs colored according to log_2_ fold-change values. miRNAs with higher cancer-associated expression changes preferentially occupy the region corresponding to reduced tissue specificity (bottom right corner), whereas miRNAs with lower or negative expression changes tend toward preserved or increased tissue specificity (top left corner). (**F**) Correlation analysis between ΔTau and log_2_ fold-change values demonstrates a strong association between changes in tissue specificity and expression alterations (Pearson’s r = 0.7; Spearman’s ρ = 0.75).

As an additional analysis of miRNA dysregulation, we compared miRNA expression between the TCGA and GTEx cohorts using the Mann–Whitney U test (FDR < 0.01, |log_2_FC| > 1). This comparison identified a substantially larger number of differentially expressed miRNAs than the recurrent dysregulation analysis: 147 miRNAs were significantly upregulated in the TCGA cohort, 91 were significantly upregulated in the GTEx cohort, and 156 showed no significant differential expression (Figure 3A). In contrast to the recurrent dysregulation analysis, this comparison revealed a much stronger separation of dysregulation classes along the ΔTau axis. miRNAs significantly upregulated in the TCGA cohort were predominantly shifted toward negative ΔTau values, indicating a loss of tissue specificity in cancer, whereas miRNAs upregulated in the GTEx cohort were enriched for positive ΔTau values, reflecting increased tissue specificity in normal tissues. In contrast, non-significant miRNAs were centered around ΔTau = 0 (Figure 3B). This pattern was further supported by classification metrics. Using a biologically defined threshold of ΔTau = 0, ΔTau demonstrated excellent discrimination between miRNAs upregulated in the TCGA and GTEx cohorts, achieving a balanced accuracy of 0.9. The difference in ΔTau distributions was highly significant (Mann–Whitney U test, *p* = 1.9 × 10^−34^) with a very large effect size (Cliff’s δ = −0.95), while the overall discriminatory performance reached a ROC-AUC of 0.97 (Figure 3C,D). In this case, the ΔGini also demonstrated a high degree of concordance with the ΔTau, with a balanced accuracy of 0.93, a Mann–Whitney U test *p*-value of 2.43 × 10^−36^, and a Cliff’s δ of −0.97 (Supplementary Figure S3D–F). This strong discriminatory performance might be explained by the relationship between expression changes and tissue specificity dynamics. A significant correlation between log_2_FC and ΔTau/ΔGini (Spearman’s ρ = −0.7 and ρ = 0.76 respectively) indicated that miRNAs with increased expression in cancer tissues tend to exhibit reduced tissue specificity (Figure 3E,F; Supplementary Figure S3G). This relationship is not unexpected, as increased tissue specificity is generally accompanied by a decrease in the overall mean expression level across tissues, whereas reduced tissue specificity reflects broader expression patterns and consequently higher mean expression. Conversely, miRNAs with higher expression in normal tissues generally showed increased tissue specificity in the cancer cohort. Importantly, this strong coupling between expression changes and tissue specificity alterations suggests that mean expression differences between TCGA and GTEx, as assessed by the Mann–Whitney U test, may partly reflect shifts in tissue specificity rather than cancer-specific regulatory alterations alone. Therefore, miRNAs supported by both approaches, recurrent dysregulation across TCGA paired projects and consistent mean expression shifts between TCGA and GTEx, were considered high-confidence dysregulated candidates.

### 2.3. Loss of Tissue Specificity and Dysregulation

**Figure 4:**
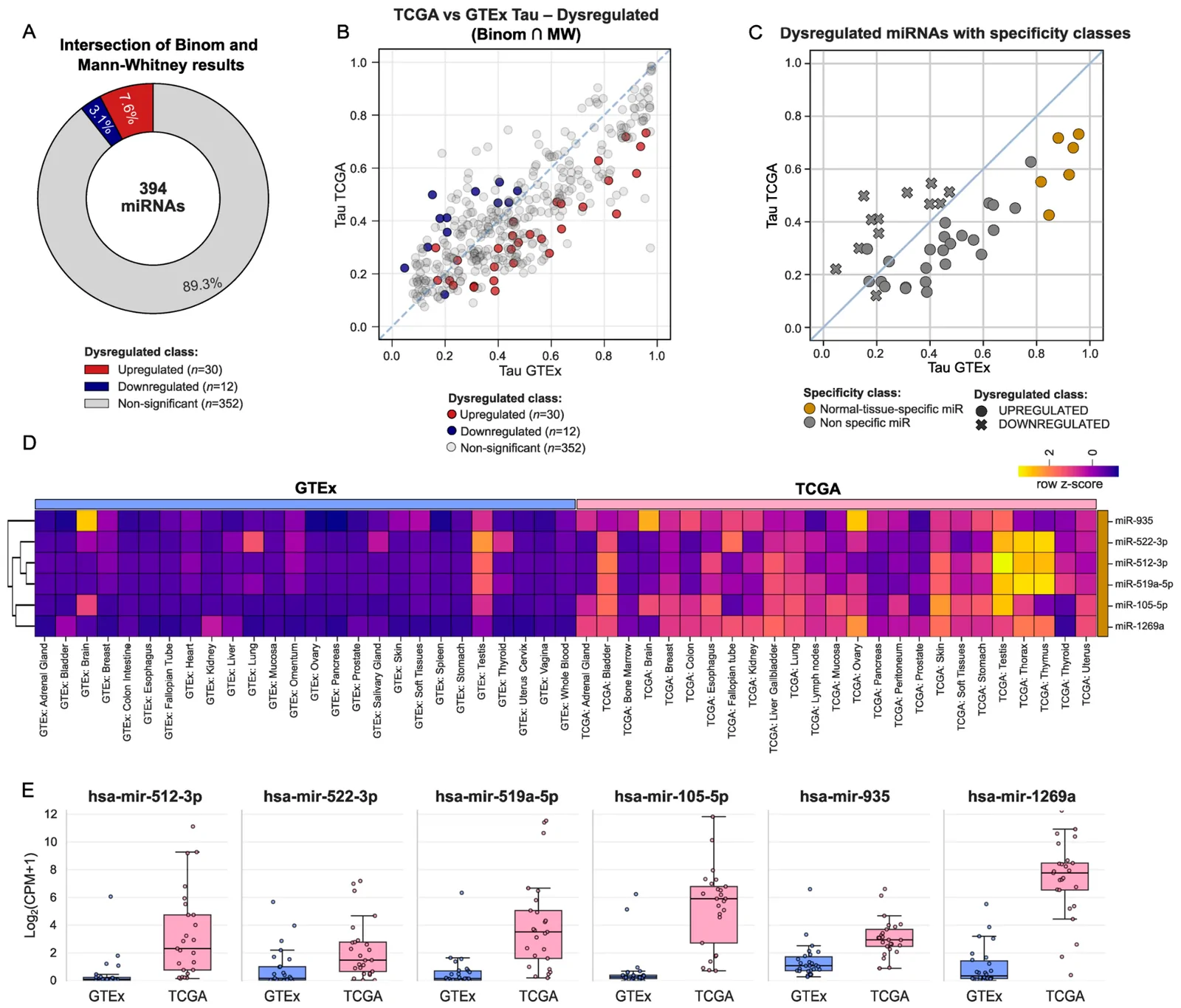
Integration of differential expression analyses and identification of miRNAs exhibiting altered tissue specificity during malignant transformation. (**A**) Distribution of miRNAs based on the intersection of two independent analyses: the directed binomial test across 17 TCGA cancer projects with matched normal tissues and comparison of mean CPM expression between GTEx and TCGA cohorts across tissue types. Among 394 analyzed miRNAs, 30 (7.6%) are consistently upregulated, 30 (3%) are consistently downregulated, and 352 (89.3%) are not significant. (**B**) Scatter plot comparing tissue specificity scores (Tau) between GTEx and TCGA cohorts. Each point represents an individual miRNA and is colored according to its dysregulation status based on the intersection of both statistical criteria. (**C**) Scatter plot highlighting dysregulated miRNAs according to their tissue specificity class. Yellow points indicate miRNAs exhibiting loss of tissue specificity during malignant transformation combined with consistent upregulation in cancer tissues, representing the primary candidates of interest. The purple cross indicates the miRNA showing acquired tissue specificity accompanied by consistent downregulation in cancer tissues. (**D**) Heatmap illustrating expression patterns of selected miRNAs with loss or acquired tissue specificity across GTEx and TCGA cohorts, demonstrating the transition of tissue expression patterns during malignant transformation. (**E**) Boxplots comparing log_2_ (CPM + 1) expression levels of selected miRNAs between GTEx and TCGA cohorts, illustrating differences in expression patterns associated with altered tissue specificity.

To identify the most robustly dysregulated miRNAs, we intersected the results of two independent analyses: (i) the directed binomial test summarizing differential expression across 17 TCGA cancer projects with matched normal tissues and (ii) the Mann–Whitney U test comparing mean miRNA expression between the GTEx and TCGA cohorts. Among the 394 analyzed miRNAs, 30 (7.6%) were consistently upregulated and 12 (3%) were consistently downregulated, whereas the remaining 352 (89.3%) did not satisfy both statistical criteria (Figure 4A,B; Supplementary Figure S4; Supplementary Table S3). From these robustly dysregulated miRNAs, we identified a biologically relevant subset: normal-tissue-specific miRNAs that were consistently upregulated in cancer (*n* = 6; miR-519a-5p, miR-512-3p, miR-522-3p, miR-105-5p, miR-935, miR-1269a) (Figure 4C).

To further investigate the functional relevance of the identified six miRNAs, pathway enrichment analysis was performed separately for each miRNA using experimentally validated and predicted target genes. Functional enrichment analysis revealed that all six miRNAs were associated with processes related to developmental processes and neurogenesis (e.g., GO:0032502, GO:0022008). Five miRNAs, miR-105-5p, miR-512-3p, miR-519a-5p, miR-522-3p, and miR-935, were associated with cell division and neuronal differentiation processes (e.g., GO:0051301, GO:0030182, GO:0048699). miR-105-5p, miR-512-3p, miR-522-3p, and miR-935 were further linked to cellular stress responses, regulation of apoptosis/programmed death (e.g., GO:0043066, GO:0043069, GO:0043067), and tube and blood vessel development. These four miRNAs were also associated with several signaling pathways involved in cell fate and survival, including cell fate commitment, MAPK, TGF-β, PDGF, and PI3K–Akt signaling. The WNT signaling pathway was associated with miR-105-5p and miR-522-3p, whereas regulation of the TP53 pathway was observed for miR-105-5p, miR-522-3p, and miR-935. Angiogenic processes, including blood vessel formation and regulation of angiogenesis (e.g., GO:0001525, GO:0002040, GO:0010596), as well as TOR and NOTCH signaling and their regulation, were predominantly associated with miR-935. Targets of miR-522-3p were associated with signaling mediated by KIT, PTK6, EGFR, and MET. In contrast, miR-512-3p showed a particularly strong association with immune system modulation, including T-cell activation and differentiation and related immune processes (e.g., GO:0035710, GO:0042093, GO:0045088). miR-1269a was associated with PTEN and BMP signaling and regulation. Finally, miR-105-5p was linked to FoxO, NTRK1, and mTOR signaling pathways, as well as processes related to stem cell maintenance (Supplementary Table S4).

## 3. Discussion

This study presents an integrative analysis of microRNA (miRNA) biology by combining tissue specificity with differential expression across healthy and malignant tissues. This approach characterizes miRNAs not only by changes in expression but also by alterations in tissue-specific regulatory programs during malignant transformation, a dimension that has received relatively little attention in previous pan-cancer studies. Tissue specificity was primarily quantified using the Tau index, a robust and widely used metric of expression specificity, while the Gini index was used as an independent validation metric to confirm the robustness of the observed patterns. To our knowledge, this is the first large-scale study to systematically examine the relationship between miRNA dysregulation and changes in tissue specificity across healthy and cancerous tissues.

In contrast to previous pan-cancer studies that primarily relied on differential expression analysis between tumor and matched normal samples to identify potential oncogenic or tumor-suppressive miRNAs [12,13,14], we incorporated normal tissue expression data from GTEx as an external replication layer to assess the robustness and consistency of our TCGA-based findings. This approach was implemented to minimize false-positive findings that may arise from recurrence-based analyses alone. A representative example is miR-671-5p. Based solely on recurrence across TCGA projects, this miRNA appeared to function as a consistent oncomiR, being upregulated in 11 of 17 cancer types and downregulated in none. However, direct comparison of global mean expression demonstrated significantly higher expression in normal tissues (GTEx) than in tumors (TCGA), consistent with previous studies describing miR-671-5p primarily as a tumor suppressor in multiple malignancies [15,16,17,18,19]. Similar discrepancies were observed for six additional miRNAs, including miR-122-5p, miR-9-3p, miR-760, miR-148b-5p, and miR-135b-5p. Conversely, three miRNAs (miR-29c-3p, miR-30a-5p, and miR-140-3p) were classified as recurrently downregulated despite exhibiting significantly higher global mean expression in the cancer cohort. Known tumor-suppressive miRNA families, including let-7 [20], miR-34 [21], and miR-200 [22], were also not significantly dysregulated in our analysis. However, the miR-200a-5p isoform met both statistical criteria (ΔTau = −0.16) and was specifically identified as upregulated oncomiR. Although the miR-200 family is well known for its role in suppressing epithelial–mesenchymal transition (EMT), recent evidence suggests a more complex role for miR-200a-5p. In vitro studies have shown that miR-200a-5p can promote migration and proliferation while suppressing apoptosis in malignant cells, and associated with poor prognosis in lung adenocarcinoma [23,24]. Other examples of miRNAs that met both criteria and have been characterized predominantly as tumor suppressors include miR-1-3p (ΔTau = +0.14) and miR-145-3p/5p (ΔTau = +0.15 and −0.08, respectively), consistent with previous reports describing their tumor-suppressive roles in cancer [25,26,27]. Well-characterized miRNAs with pro-oncogenic properties that also showed a consistent cancer-upregulated signal in our analysis included miR-21-3p (ΔTau = −0.25) [28,29], miR-20a-5p (ΔTau = +0.13) [30], miR-183-5p (ΔTau = −0.16), and miR-106b-3p (ΔTau = −0.21) [31]. Although the requirement for concordance between two independent criteria inevitably results in the exclusion of some potentially relevant oncomiRs and tumor-suppressive miRNAs, our analysis indicates that this trade-off improves the reliability of the resulting miRNA set by reducing false-positive signals inherent to bioinformatic analyses. This two-step validation strategy therefore allowed us to define a high-confidence set of cancer-associated miRNAs.

Our results suggest that changes in tissue specificity are associated, at least in part, with miRNA dysregulation. This observation is biologically plausible, as miRNAs involved in tissue-specific functions may acquire more widespread activity during malignant transformation and contribute to common oncogenic processes. Interestingly, our finding that miRNAs with high tissue specificity in normal tissues tend to lose this specificity in cancer, as reflected by an inverse association between normal-tissue specificity and Δspecificity, is partially consistent with findings from a similar analysis of protein-coding transcripts based on GTEx and TCGA data. In that study, cancer was characterized by the activation of genes not normally expressed in the tissue of origin, as well as by the upregulation of genes normally specific to other tissues [32]. From a therapeutic perspective, such miRNAs may represent attractive candidates for anti-miRNA strategies, since their limited expression in normal tissues could reduce off-target effects associated with systemic inhibition. We identified six miRNAs that loss tissue specificity and displayed robust upregulation pattern in cancer. The functional relevance of this subset is supported by published experimental evidence. Six miRNAs within this signature have documented oncogenic activity across multiple malignancies. MiR-522-3p promotes proliferation in osteosarcoma [33] and enhances tumor growth, metastasis, and epithelial–mesenchymal transition (EMT) in colorectal cancer [34,35]. Although evidence for miR-519a-5p remains limited, it has been reported to increase the migratory and invasive capacity of hepatocellular carcinoma cells [36], whereas an opposite effect, namely inhibition of the Warburg phenotype, has been described in ovarian cancer [37]. MiR-105-5p has also been consistently implicated in tumor progression by promoting EMT and immunosuppression in bladder [38], breast [39], and gastric cancers [40]. Similarly, miR-1269a exhibits predominantly oncogenic activity, promoting EMT in colorectal and bladder cancers [41,42], enhancing migration and proliferation in esophageal squamous cell carcinoma [43], and stimulating glioma progression while suppressing apoptosis in vitro [44]. In contrast, miR-512-3p and miR-935 display context-dependent functions. MiR-512-3p has been associated with poor clinical outcomes in pediatric medulloblastoma [45] and shown to activate the PI3K/AKT and Wnt/β-catenin signaling pathways in colorectal cancer [46]. It has also been implicated in the regulation of chemoresistance in ovarian cancer through the miR-512-3p/RPS6KA2 axis. Conversely, other studies reported that the miR-512-3p/HK-2 axis suppresses tumor progression in ovarian [47], prostate [48], and colorectal cancers [49]. Likewise, miR-935 has been reported to stimulate proliferation in hepatocellular and gastric cancers and to promote chemoresistance in lung cancer through the miR-935/SOX7 axis [50,51,52]. Similar oncogenic effects have also been described in choriocarcinoma via the miR-935/GJA1 axis [53]. In contrast, the miR-935/HIF1α axis has been shown to suppress glioma progression [54], and miR-935 inhibits proliferation, migration, and invasion in oral squamous cell carcinoma [55].

Although clinical experience with miRNA inhibitors (antagomirs/anti-miRs) in oncology remains limited to early-phase trials, available evidence indicates a generally favorable safety profile. Cobomarsen (MRG-106), an LNA inhibitor of miR-155, was well tolerated in Phase 1, while the subsequent Phase 2 SOLAR trial was terminated for organizational reasons unrelated to safety or efficacy (NCT02580552, NCT03713320). Likewise, the first-in-class LNA-i-miR-221 demonstrated no grade 3–4 toxicities and preliminary antitumor activity in a first-in-human Phase 1 study [56]. In contrast, clinical development of the miR-34a mimic MRX34 was discontinued because of severe immune-mediated toxicity [57,58]. Notably, both miR-155-5p and miR-10b exhibit low tissue specificity and are broadly expressed in healthy tissues, yet their pharmacological inhibition has remained clinically tolerable. Together, these findings support the potential of targeting oncogenic miRNAs and suggest that candidates with higher baseline tissue specificity, such as those identified in this study, may have favorable characteristics for therapeutic development. Notably, miR-155-5p and both miR-10b isoforms, which have already entered clinical development as therapeutic targets, exhibit relatively low tissue specificity and are broadly expressed in healthy tissues. Despite this, inhibition of these miRNAs has shown acceptable tolerability in clinical studies. In comparison, the candidates identified in our study combine broad upregulation across multiple malignancies with substantially higher tissue specificity under normal physiological conditions. Although this study is based exclusively on bioinformatic analyses and this hypothesis requires thorough experimental validation to assess both therapeutic efficacy and safety, the combination of broad cancer-associated upregulation and restricted expression in normal tissues provides a rationale for further investigating whether therapeutic inhibition of these miRNAs may offer a favorable therapeutic index while targeting molecular pathways shared across multiple cancer types.

Several limitations should be considered when interpreting these findings. First, the tissue compositions of the TCGA and GTEx cohorts differ, despite having comparable numbers of tissue types (25 and 26, respectively). For instance, TCGA includes tissues such as bone marrow, lymph nodes, peritoneum, thorax, and thymus, which are not represented in GTEx. Conversely, GTEx contains tissues including the heart, omentum, salivary gland, spleen, vagina, and whole blood, which are absent from TCGA. These differences in tissue representation may affect the assessment of tissue specificity and potentially influence the observed results. Although the concordance between the TCGA- and GTEx-derived results provides supportive evidence for the robustness of our findings, these datasets were generated from distinct cohorts using different experimental and tissue-processing protocols. Therefore, potential dataset-specific and batch-related effects cannot be completely excluded. Accordingly, the GTEx analysis should be interpreted as supportive external replication rather than fully independent validation of the TCGA-based findings.

## 4. Materials and Methods

### 4.1. Data Preprocessing and Inclusion Criteria

MiRNA expression profiles from the healthy (GTEx) [59] and tumor (TCGA, TARGET, CGCI, CPTAC) tissue [60] databases were used in this study. During preprocessing, samples were grouped according to normal tissue types and cancer tissue types. Only tissues represented by at least 10 biological samples were included in the analysis. To ensure comparability between normal and tumor datasets, tissue categories were harmonized based on the closest possible correspondence in both tissue type and number of represented tissues. As a result, 26 tissue types from GTEx and 25 tissue types from TCGA were included in the comparative analysis.

MiRNAs with a maximum tissue expression level below 20 counts per million (CPM) were filtered out separately for the GTEx and TCGA datasets. Subsequent analyses were restricted to miRNAs detected in both datasets. Following these filtering steps, the initial sets of 2011 miRNAs from TCGA and 827 miRNAs from GTEx were reduced to 394 miRNAs retained for further analysis.

### 4.2. Tissue Specificity Analysis

The degree of tissue-specific expression for each miRNA was quantified using the Tau (τ) and Gini tissue specificity indices [8,9]. Tau and Gini values were calculated based on the log_2_-transformed mean miRNA expression levels across tissue groups after CPM normalization, with a pseudocount of 1 added to avoid zero values. The Tau index was calculated according to the following formula:

$$\tau =\frac{\sum_{i=1}^{n}\;\left(1-{\hat{x}}_{i}\right)}{n-1}$$

where *n* denotes the total number of groups, and ${\hat{x}}_{i}=\frac{x_{i}}{max(x)}$ represents the normalized expression of a given miRNA in the *i*-th group relative to its maximum expression value across all groups.

The Gini index was calculated using the following formula:

$$\frac{n+1}{n}-\;\frac{2\sum_{i=1}^{n}\;\left(n+1-i\right)x_{i}}{n\sum_{i=1}^{n}\;x_{i}}$$

Tau and Gini values range from 0 (ubiquitous expression) to 1 (strict tissue specificity). The indices were calculated independently for the healthy and cancer cohorts using specificity-based classification. Of the two specificity indices considered, Tau and Gini, the Tau index was selected for subsequent analyses (see Section 2). A specificity threshold of 0.8 was applied for the Tau index, in accordance with the previously published literature [9]. The choice of this threshold was further supported by an analysis of result interpretability (see Section 2). Using a threshold of τ > 0.8, all analyzed miRNAs were classified into four functional categories according to their tissue specificity patterns in normal and tumor tissues:

1. Normal-tissue-specific miRs. miRNAs exhibiting tissue specificity only in normal tissues ($\tau_{GTEx}\geq 0.8,\;\tau_{TCGA}<0.8)$.
2. Cancer-tissue-specific miRs. miRNAs exhibiting tissue specificity exclusively in cancer tissues ($\tau_{TCGA}\geq 0.8,\;\tau_{GTEx}<0.8)$.
3. Tissue specific miRs. miRNAs retaining high tissue specificity in both datasets (τ≥0.8 in both cohorts).
4. Non-specific miRs. miRNAs exhibiting non-specific expression patterns in both normal and tumor conditions (τ<0.8).

### 4.3. Statistics

For each miRNA, paired Tau (or Gini) values were obtained in GTEx and TCGA, and the change in specificity was defined as Δ = index_TCGA − index_GTEx. Distributions of specificity indices were compared between cohorts with a two-sided Mann–Whitney U test, whereas overall directional change was assessed with a one-sided Wilcoxon signed-rank test on Δ (H_1_: median Δ < 0). The same paired Wilcoxon test, together with a one-sided binomial test for enrichment of points below versus above the diagonal (H_1_: P(TCGA < GTEx) > 0.5), was applied within GTEx-index quartiles and within the high-specificity subsets defined by cohort-specific thresholds (GTEx ≥ threshold and TCGA ≥ threshold). Discordant categorical calls (normal-tissue-specific versus cancer-tissue-specific) were likewise compared with a one-sided binomial test against equal probability. Association between specificity loss and baseline normal-tissue specificity was quantified by Spearman correlation between Δ and the GTEx index. Finally, ordinary least-squares regression of the TCGA index on the GTEx index was used to test whether the fitted slope was significantly less than one (one-sided *t*-test), consistent with systematic attenuation of tissue specificity in tumors.

### 4.4. Differential Expression Analysis Within TCGA Cohort and Recurrent Dysregulation Analysis

To identify dysregulated miRNAs, expression levels between tumor and normal tissues were compared within each cancer project containing paired controls in TCGA dataset. A total of 17 projects were included, meeting the criterion of ≥ 10 normal samples and ≥10 tumor samples. Differential expression analysis was performed using the PyDESeq2 library (version 0.5.4) [61]. Model parameters were estimated using DeseqDataSet and DeseqStats with refit_cooks = True to minimize the influence of outliers. The comparison was performed for tumor vs. normal, and results were considered statistically significant at an adjusted *p*-value < 0.01 and |log_2_FC| > 1.

To assess whether individual miRNAs exhibited a significant bias toward upregulation or downregulation across cancer projects, we applied a one-sided exact binomial test. For each miRNA, the number of significantly upregulated ($n_{up}$) and downregulated ($n_{down}$) instances was counted based on the thresholds defined above (with the direction determined by the sign of the log_2_FC), and the total number of dysregulated cases was defined as: $n_{total}=n_{up}+n_{down}$. Non-significant cases were intentionally excluded from $n_{total}$ to maintain statistical power and account for the heterogeneous nature of cancers. Under the null hypothesis, upregulation and downregulation were assumed to be equally likely among significantly dysregulated cases (H_0_: $p=\frac{1}{2};\;$where *p* denotes the probability of upregulation). Two one-sided exact binomial tests were performed: alternative = “greater” to test whether upregulated events occurred more frequently than expected by chance, and alternative = “less” to test whether downregulated events occurred more frequently than expected by chance. Statistical significance was defined as *p* < 0.05.

### 4.5. Comparison of Mean miRNA Expression Between GTEx and TCGA Tissues

As an additional criterion, mean miRNA expression levels were compared between tissues from the GTEx and TCGA cohorts. Differences in miRNA expression across tissues between the two cohorts were assessed using the Mann–Whitney rank-sum test applied to log_2_(CPM + 1) transformed expression values, followed by Benjamini–Hochberg correction for multiple testing (false discovery rate, FDR). The effect size was estimated using log_2_ fold change calculated from the mean expression values between the two cohorts. Statistical significance was defined as FDR < 0.01 combined with an absolute log_2_FC value greater than 1 (|log_2_FC| > 1).

### 4.6. Functional Analysis of miRNA’s Targets

Target genes for each miRNA were obtained from the miRTargetLink (version 2.0) portal [62], which integrates experimentally validated miRNA–target interactions from miRTarBase [63] and miRATBase (https://ccb-web.cs.uni-saarland.de/miratbase, accessed on 10 September 2026), as well as predicted target interactions compiled from miRDiP [64] and miRDB [65].

Functional enrichment analysis of miRNA target genes was performed using the STRING database (version 12.5) [66] via its API. For each miRNA, a list of predicted or experimentally supported target genes was analyzed independently. Duplicate and missing gene identifiers were removed prior to analysis. Enrichment analysis was performed for Gene Ontology Biological Process (GO BP), Kyoto Encyclopedia of Genes and Genomes (KEGG), and Reactome pathway categories. Statistical significance was assessed using the false discovery rate (FDR), and terms with FDR ≤ 0.05 were considered significantly enriched. For each enriched term, the enrichment magnitude was additionally quantified as the ratio between the proportion of genes associated with the term in the analyzed target list and the corresponding proportion in the STRING background. Enrichment results were ranked by FDR within each annotation category.

## Data Availability

The data analyzed in this study are publicly available through The Cancer Genome Atlas (TCGA) program and can be accessed via the cBioPortal for Cancer Genomics platform (https://www.cbioportal.org/, accessed on 10 September 2026). GTEx expression data are publicly available through GTEx Portal (https://gtexportal.org/home/, accessed on 10 September 2026). The code used to perform the analyses and generate the figures presented in this study is publicly available at GitHub: https://github.com/neuropromotion/mirna-specificity-analysis, accessed on 10 September 2026.

## Supplementary Materials

The following supporting information can be downloaded at https://www.mdpi.com/article/10.3390/ijms27188153/s1.
